# Assessing Predation Risk to Threatened Fauna from their Prevalence in Predator Scats: Dingoes and Rodents in Arid Australia

**DOI:** 10.1371/journal.pone.0036426

**Published:** 2012-05-01

**Authors:** Benjamin L. Allen, Luke K.-P. Leung

**Affiliations:** School of Agriculture and Food Sciences, The University of Queensland, Queensland, Australia; Australian Wildlife Conservancy, Australia

## Abstract

The prevalence of threatened species in predator scats has often been used to gauge the risks that predators pose to threatened species, with the infrequent occurrence of a given species often considered indicative of negligible predation risks. In this study, data from 4087 dingo (*Canis lupus dingo* and hybrids) scats were assessed alongside additional information on predator and prey distribution, dingo control effort and predation rates to evaluate whether or not the observed frequency of threatened species in dingo scats warrants more detailed investigation of dingo predation risks to them. Three small rodents (dusky hopping-mice *Notomys fuscu*s; fawn hopping-mice *Notomys cervinus*; plains mice *Pseudomys australis*) were the only threatened species detected in <8% of dingo scats from any given site, suggesting that dingoes might not threaten them. However, consideration of dingo control effort revealed that plains mice distribution has largely retracted to the area where dingoes have been most heavily subjected to lethal control. Assessing the hypothetical predation rates of dingoes on dusky hopping-mice revealed that dingo predation alone has the potential to depopulate local hopping-mice populations within a few months. It was concluded that the occurrence of a given prey species in predator scats may be indicative of what the predator ate under the prevailing conditions, but in isolation, such data can have a poor ability to inform predation risk assessments. Some populations of threatened fauna assumed to derive a benefit from the presence of dingoes may instead be susceptible to dingo-induced declines under certain conditions.

## Introduction

The prevalence of prey remains in predator scats (or faeces) is most often used to investigate predator diets [1], [2], although the same data can also be used to assess the distribution of rare or cryptic species (e.g. [3], [4]). In turn, predator scat data is also commonly used to gauge the risk of predation to species of conservation significance (e.g. [5], [6]). However, the reliability of scat data used for this purpose is questionable [7], and is made more difficult by the inability of scat data to make reliable inferences about what a predator did, does or could eat at other times and places [8]. Understanding the limitations and uses of predator scat data is therefore important for formulating appropriate management strategies for predators and prey in places where predation is considered an important risk factor for threatened species.

Australia has a unique and diverse assemblage of endemic native fauna, although many of these species are now either extinct, rare or in decline [9]–[11]. Post-European impacts associated with the introduction of pastoralism (i.e. livestock grazing and waterpoint establishment), rabbits *Oryctolagus cuniculus*, red foxes *Vulpes vulpes* and feral cats *Felis catus* have been particularly significant factors in the demise of many species (e.g. [12], [13]). These (and other) factors can operate in concert whereby exotic herbivores deplete the food and shelter available to native species, which is then followed by severe predation from native and introduced predators [14], [15]. Dingoes (*Canis lupus dingo* and other wild-living *Canis*) have also been implicated in the declines of several native fauna (e.g. [14], [16]–[18]), although their direct impacts are often presumed to be of lesser importance than their indirect benefits [19], [20]. This may yet prove true in some cases, but the direct risk of dingoes to locally threatened populations of native fauna may still be important regardless of any indirect benefits their presence might provide [7], [21].

Dingoes are a charismatic and iconic terrestrial predator associated with Australian wilderness areas. They presently occupy top-predator status and are ubiquitous across all mainland biomes, though their densities vary between regions [22], [23]. Their derivation from gray wolves *Canis lupus* and their long history of domestication [21], [24], [25] means that modern dingoes are generalist predators that consume prey species ranging from insects to water buffalo *Bubalis bubalus* across their extended range ([16]; and studies listed in Table S1). Dingoes have been implicated in the declines of large, medium and small prey species historically and in the recent past ([14], [16], [26], but see [17], [18], [27] for specific examples). Predation by dingoes and other wild dogs has also been recognised as a known or potential threat to at least 14 endangered vertebrates nationally for species weighing as little as 70 g (Table 1). Some studies (e.g. [28]) have predicted that several wild mammal species in arid areas are likely to increase in the absence of dingoes, and others [29] report the outcome of a failed burrowing bettong *Bettongia leseuer* reintroduction attempt in northern South Australia (NSA) where 14 of the 101 bettongs released were killed by undetectably low populations of dingoes within 24 hrs, the rest succumbing to predation by unidentified predators within a few months. Dingo predation has also been predicted to threaten up to 94% of listed threatened mammals, birds and reptiles in arid and semi-arid areas ([7], but see also [30]). Given these broad predictions of risk and the knowledge that dingoes can exploit small prey species under certain conditions (e.g. [31]–[34]), it seems prudent to evaluate the potential threat dingoes pose to local populations of threatened prey species known to be eaten by dingoes.

**Table 1:** Threatened species listed in the Australian *Environment Protection and Biodiversity Conservation Act 1999* that are known or potentially threatened by dingoes and other wild dogs, as identified in their recovery plans (from www.environment.gov.au, accessed 15^th^ December 2011).

Species type	Common name	Scientific name	Adult weight (g)
Mammal	Marsupial moles	*Notorycetes typhlops*, *N. caurinus*	70
Mammal	Smoky mouse	*Pseudomys fumeus*	86
Bird	Black-breasted button-quail	*Turnix melanogaster*	100
Mammal	Golden bandicoot	*Isoodon auratus*	670
Mammal	Northern quoll	*Dasyurus hallucatus*	1,200
Mammal	Greater bilby	*Macrotis lagotis*	2,500
Mammal	Long-footed potoroo	*Potorous longipes*	2,500
Bird	Mallefowl	*Leipoa ocellata*	2,500
Mammal	Bridled nailtail wallaby	*Onychogalea fraenata*	8,000
Mammal	Proserpine rock-wallaby	*Petrogale persephone*	8,800
Mammal	Koala	*Phascolarctos cinereus*	12,000
Mammal	Northern hairy-nosed wombat	*Lasiorhinus krefftii*	31,000
Bird	Southern cassowary	*Casuarius casuarius johnsonii*	60,000
Reptile	Marine turtles (eggs and hatchlings)	Various	-

Information from the contents of ∼32,000 dingo scats and stomachs collected from across Australia since the late 1960s provide the foundation of our current understanding of the prey important to dingoes. Almost half (n = 12,802) of these records collected prior to the turn of the century have already been summarised [16], while the remainder are scattered throughout various published and unpublished reports (Table S1). Information from arid areas comprises about 32% of the available literature (inclusive of the present study), though data from NSA is limited. Cupples et al. [35] and Letnic et al. [28] together presented the results of 597 dingo scats collected from the Strzelecki Desert, reporting that dingoes have a high degree of dietary overlap with foxes and cats. Wallach et al. [36] and Wallach and O'Neill [37] report the collection of over 900 dingo scats from South Australia, though information on dingo diets from almost all of these scats appears unavailable. No other information on dingo diets from South Australia appears available (Table S1).

This study uses dingo scat data from a large-scale manipulative experiment on dingo ecology in the arid zone of NSA [38] to determine the prevalence of threatened fauna in dingo scats. For each threatened species detected in scats, available additional information was subsequently used to explore the potential roles dingoes may have on the persistence of the species and the reliability of scat data for making predictions about predation risks to these species. The intention is not to demonstrate that dingoes *do* present a risk to threatened species, but rather to assess the possibility that dingoes *could* present a risk under future conditions.

## Methods

### Study sites

**Figure 1:**
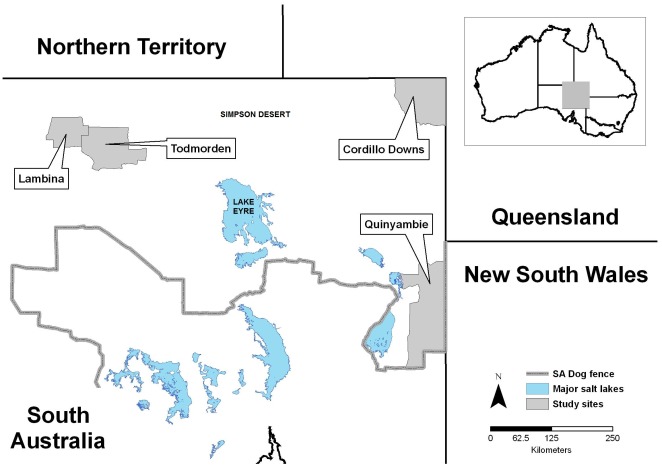
Location of study sites.

The study was conducted in beef cattle production regions north of the interstate dingo barrier fence (known as ‘the dog fence’), which was erected to facilitate the eradication of dingoes in sheep production regions to the south in the early 1900s [39]. For management purposes, the area north of the fence in NSA is divided into the ‘northeast pastoral zone’ and the ‘northwest pastoral zone’ [38] which are broadly separated by Lake Eyre and the Simpson Desert. Scat collection took place on five cattle stations within these zones, with Quinyambie and Cordillo Downs in the northeast, and Todmorden, Lambina and Hamilton in the northwest (Fig. 1).

Quinyambie Station is located in the sandy Strzelecki Desert, has a mean annual rainfall (MAR) of ∼160 mm, and is comprised of parallel sand dunes dominated by hopbush *Dodonaea viscosa*, buckbush *Salsola kali*, and a variety of grasses and burrs including kerosene grass *Aristida* spp. and copperburr *Sclerolaena* spp. [40]. Cordillo Downs Station is in the extreme northeast of South Australia inside Haddon Corner, receives a MAR of ∼167 mm, and incorporates both large, irregular sand dunes and extensive stony gibber plains. These contain beefwood *Grevillea striata* and spinifex *Triodia* spp. on the dunes, Mitchell grass *Astrebla* spp. on the plains, and red gum *Eucalyptus camaldulensis* and mineritchie *Acacia cyperophylla* in the drainage lines. Todmorden, Hamilton and Lambina Stations adjoin each other, and are located around the sandy Pedirka Desert in the central-north of the state, have a MAR of ∼180 mm, and are comprised of sandy deserts dominated by mulga *Acacia aneura* stands, with stony plain and drainage line vegetation similar to Cordillo Downs Station. Rainfall means were derived from long-term Bureau of Meteorology (www.bom.gov.au) data collected from nearby weather stations at Birdsville (since 1892), Frome Downs Station (since 1889), Hamilton Station (since 1884), Innamincka (since 1882), Macumba Station (since 1891) and Todmorden Station (since 1949). Information on South Australian floral species was obtained from [40].

Although large, medium and small prey were present at each site, the relative abundance and availability of each species was not consistent between sites and varied throughout the study period (B. Allen, unpublished data). Most of the mammalian prey species identified in dingo scats were present at each site. However, feral pigs are found only on Cordillo Downs [23] and some of the small mammals have restricted distributions [11] and are likely to be rare or absent at some sites [3]. Macropods are uncommon at Quinyambie, relatively common at Cordillo Downs and abundant at the other three sites [41]–[43]. Rabbits are abundant at Quinyambie and uncommon at the other four sites. Further information on the distribution of native and introduced prey species can be found elsewhere (e.g. [11], [23], [44], [45]).

### Scat analysis

Dingo scats were distinguished from those of other predators based on their size, shape, smell and placement [46], and were collected during repeated visits to the stations between May 2008 and December 2010. Scat collection occurred once at Hamilton, five times at Lambina, six times at Cordillo Downs, eight times at Quinyambie and nine times at Todmorden during this period. Because of the high abundance (and thousands of available scats) of dingoes at Quinyambie [38], [47], scat collection was restricted to discrete, fenced (to exclude cattle) areas around five permanent artificial livestock watering points. At the other four sites, scats were collected from a wide variety of waterpoints, vehicle tracks, dry creek crossings, intersections and other locations where dingoes were expected to defecate more frequently.

Dingo scats collected were sterilised and washed by a professional service provider (B. Triggs, Mallacoota, Victoria) who then searched each scat for the remains of individually identifiable mammal species using established methods (described in [48]). Results were reported at the genus level (or higher) where there was ambiguity over positive species-level identification. Each terrestrial mammal detected was categorised as a small, medium or large mammal using five alternative body weight classes reported by [16], [28], [49], [50] and [35] (but sourced from [4]). Non-mammal food items were categorised simply as birds, reptiles (inclusive of both smooth- and rough-scaled species), invertebrates or vegetation, which were only described to the species level opportunistically (by staff at the South Australian museum) according to the incidental presence of diagnostic bones and other features (such as teeth or scales) in the scat. Threatened species were identified from lists in the South Australian *National Parks and Wildlife Act 1974* and the Australian *Environment Protection and Biodiversity Conservation Act 1999*. Results are expressed as the ‘percent occurrence in scats’ because our study was primarily concerned with the presence of infrequently detected prey species in dingo scats and not dingo diet per se [1].

## Results

A total of 4087 dingo scats were collected from all sites during the study (Table 2). Information from these scats represents ∼40% of the literature from arid areas or ∼13% of the entire available literature on dingo scats and stomachs from across Australia (Tables S1 and S2). The majority of scats were collected from Quinyambie (n = 2263) and Cordillo Downs (n = 1303), with Todmorden, Lambina and Hamilton yielding fewer scats (n = 424, 79 and 18 respectively). Seventeen mammal species were detected (Table 2), inclusive of both dusky hopping-mice *Notomys fuscus* and fawn hopping-mice *N. cervinus* (the vast majority of which were *N. fuscus*; [3]) here grouped at the genus level. Mammals were the most frequently occurring taxa overall, although reptiles, invertebrates and/or vegetation occurred relatively frequently in scats from some sites (Table 2). Incidental identification of other animals detected several birds, reptiles and one amphibian in dingo scats (Table 3). Of these, bearded dragons *Pogona barbata* and spiny-tailed skinks *Egernia stokesii* appeared most common.

**Table 2:** The percent occurrence of prey remains found in 4087 dingo scats from five sites in northern South Australia between March 2008 and December 2010.

		*N* scats containing each item (% occurrence)					
Common name	Taxonomic name	Cordillo Downs	Hamilton	Lambina	Quinyambie	Todmorden	Total
Bone fragments only	.	54 (4.14)	3 (16.67)	8 (10.13)	68 (3.00)	40 (9.43)	**173 (4.23)**
Cattle	*Bos taurus*	311 (23.87)	8 (44.44)	30 (37.97)	162 (7.16)	164 (38.68)	**675 (16.52)**
Dingo	*C. l. dingo* (grooming)	55 (4.22)	0 (0.00)	3 (3.80)	90 (3.98)	23 (5.42)	**171 (4.18)**
Dingo	*C. l. dingo* (prey)	8 (0.61)	0 (0.00)	0 (0.00)	18 (0.80)	3 (0.71)	**29 (0.71)**
Feral cat	*Felis catus*	5 (0.38)	0 (0.00)	0 (0.00)	4 (0.18)	1 (0.24)	**10 (0.24)**
Human	*Homo sapiens*	2 (0.15)	0 (0.00)	0 (0.00)	0 (0.00)	0 (0.00)	**2 (0.05)**
Euro	*Macropus robustus*	0 (0.00)	0 (0.00)	0 (0.00)	0 (0.00)	2 (0.47)	**2 (0.05)**
Red kangaroo	*Macropus rufus*	50 (3.84)	2 (11.11)	16 (20.25)	43 (1.90)	150 (35.38)	**261 (6.39)**
Lesser long-eared bat	*Nyctophilus geoffroyi*	0 (0.00)	0 (0.00)	0 (0.00)	1 (0.04)	0 (0.00)	**1 (0.02)**
House mouse	*Mus musculus*	199 (15.27)	0 (0.00)	10 (12.66)	40 (1.77)	12 (2.83)	**261 (6.39)**
No identifiable hair	.	94 (7.21)	1 (5.56)	5 (6.33)	89 (3.93)	17 (4.01)	**206 (5.04)**
Hopping-mouse	*Notomys* spp.	74 (5.68)	0 (0.00)	2 (2.53)	192 (8.48)	16 (3.77)	**285 (6.97)**
Rabbit	*Oryctolagus cuniculus*	273 (20.95)	4 (22.22)	9 (11.39)	1745 (77.11)	35 (8.25)	**2066 (50.55)**
Plains mouse	*Pseudomys australis*	0 (0.00)	0 (0.00)	0 (0.00)	1 (0.04)	0 (0.00)	**1 (0.02)**
Sandy inland mouse	*Pseudomys hermannsburgensis*	1 (0.08)	0 (0.00)	0 (0.00)	0 (0.00)	0 (0.00)	**1 (0.02)**
Forrest's mouse	*Leggadina forresti*	1 (0.08)	0 (0.00)	0 (0.00)	0 (0.00)	0 (0.00)	**1 (0.02)**
Long-haired rat	*Rattus villosissimus*	183 (14.04)	0 (0.00)	0 (0.00)	8 (0.35)	0 (0.00)	**191 (4.67)**
Fat-tailed dunnart	*Sminthopsis crassicaudata*	1 (0.08)	0 (0.00)	0 (0.00)	2 (0.09)	0 (0.00)	**3 (0.07)**
Stripe-faced dunnart	*Sminthopsis macroura*	142 (10.90)	1 (5.56)	0 (0.00)	23 (1.02)	1 (0.24)	**167 (4.09)**
Feral pig	*Sus scrofa*	3 (0.23)	0 (0.00)	0 (0.00)	0 (0.00)	0 (0.00)	**3 (0.07)**
Echidna	*Tachyglossus aculeatus*	0 (0.00)	0 (0.00)	0 (0.00)	0 (0.00)	2 (0.47)	**2 (0.05)**
Invertebrates	.	172 (13.20)	2 (11.11)	6 (7.59)	314 (13.88)	29 (6.84)	**523 (12.80)**
Vegetation	.	308 (23.64)	3 (16.67)	27 (34.18)	332 (14.67)	75 (17.69)	**745 (18.23)**
Birds	.	61 (4.68)	1 (5.56)	6 (7.59)	112 (4.95)	15 (3.54)	**195 (4.77)**
Reptiles	.	122 (9.36)	0 (0.00)	0 (0.00)	100 (4.42)	28 (6.60)	**250 (6.12)**
Other	.	3 (0.23)	0 (0.00)	0 (0.00)	2 (0.09)	0 (0.00)	**5 (0.12)**
	***Total number of scats***	***1303***	***18***	***79***	***2263***	***424***	***4087***

**Table 3:** Incidental records of non-mammal prey from dingo scats collected from five sites in northern South Australia between March 2008 and December 2010.

Common name	Taxonomic name	Taxa	Site
Trilling frog	*Neobatrachus centralis*	Amphibian	Quinyambie
Emu	*Dromaius novaehollandiae*	Bird	Quinyambie
Galah	*Cacatua roseicapilla*	Bird	Todmorden
Shingleback	*Tiliqua rugosa*	Reptile	Quinyambie
Bearded dragon	*Pogona vitticeps*	Reptile	Cordillo Downs
Spiny-tailed skink	*Egernia stokesii*	Reptile	Cordillo Downs

The assignment of terrestrial mammal prey to small, medium or large species was consistent between all five body weight classifications (Table 4), although strict adherence to the classes originally proposed in [4] and later adopted in [35] would have classified dingoes, cats and rabbits as large prey in the present study. Dingo scats from Cordillo Downs, Hamilton, Lambina, Quinyambie and Todmorden showed that remains of small-sized mammals weighing <500 g occurred in 46%, 6%, 15%, 12% and 7% of scats respectively (22% of scats overall), with long-haired rats *Rattus villosissimus* being the largest species (at 156 g) in this category (Table 4). The smallest medium-sized mammal found in dingo scats was rabbits, and feral cats were the only medium-sized predator detected in dingo scats (Table 4). Cats, rabbits and echidnas *Tachyglossus aculeatus* were the only species within the Critical Weight Range (35 g–5500 g; CWR; [51]) that were not included in our classification of small mammals <500 g.

**Table 4:** Body weight classifications for the terrestrial mammals identified in dingo scats from northern South Australia between March 2008 and December 2010.

	Adult weight (g)	Corbett 2001	Cupples et al. 2011	Letnic et al. 2009	Burnett 1995	Glen & Dickman 2008
Small/medium body weight class cut-offs		500 g/15,000 g	100 g/999 g	1,000 g/10,000 g	500 g/2,000 g	499 g/6,999 g
*Bos taurus*	600,000	Large	Large	Large	Large	Large
*C. l. dingo* (prey)	15,000	Large	Medium*	Large	Large	Large
*Felis catus*	5,000	Medium	Medium*	Medium	Large	Medium
*Macropus robustus*	30,000	Large	Large	Large	Large	Large
*Macropus rufus*	35,000	Large	Large	Large	Large	Large
*Mus musculus*	20	Small	Small	Small	Small	Small
*Notomys* spp.∧	32	Small	Small	Small	Small	Small
*Oryctolagus cuniculus*	1,500	Medium	Medium*	Medium	Medium	Medium
*Pseudomys australis*∧^#^	40	Small	Small	Small	Small	Small
*Rattus villosissimus*	156	Small	Small	Small	Small	Small
*Sminthopsis crassicaudata*	15	Small	Small	Small	Small	Small
*Sminthopsis macroura*	20	Small	Small	Small	Small	Small
*Sus scrofa*	120,000	Large	Large	Large	Large	Large
*Tachyglossus aculeatus*	5,000	Medium	Medium*	Medium	Large	Medium

∧ *Listed threatened species.*

* *Species discussed in original studies as ‘medium-sized’ despite weighing >999 g.*

No threatened birds or reptiles were detected in our incidental identification of these taxa (Table 3) and the only three listed threatened mammals detected in dingo scats were fawn hopping-mice, dusky hopping-mice and plains mice *Pseudomys australis* (Table 4). Plains mice were detected only once from Quinyambie and fawn hopping-mice were also detected infrequently from Cordillo Downs and Quinyambie [3]. Of the 2263 scats collected from Quinyambie, 192 scats (8%) contained hopping-mice (predominately *N. fuscus*; [3]) and 1745 scats (77%) contained rabbits. Of the scats containing hopping-mice, 120 of them (63%) contained hopping-mice as the sole vertebrate prey item. Of the 33 scats containing hopping-mice and another vertebrate prey item, 30 of them (91%) were second (in volume) to rabbits.

## Discussion

Dingoes are atypical apex predators predisposed to present direct risks to CWR species [21] which can face significant risk of predation from cats, foxes and dingoes alike [7], [52]. However, populations of smaller species (<35 g) can also be threatened by these predators (e.g. [33], [53]). It is not surprising then that a variety of small mammals were detected in dingo scats from all sites and occurred in up to 46% of scats (Table 2). Despite the various body weight classes used to define ‘small mammals’, the consistency between them (Table 4) is probably due to the relative absence of extant mammals within the CWR [11], [51]. Thus, arbitrary selection of a cut-off value to differentiate between small and medium prey weighing anywhere between 40 g and 1500 g would only make a difference to one extant mammal detected in scats (*R. villosissimus*), suggesting that the adoption of any of the published classes are sufficient to reliably categorise prey in to body weight classes. No large or medium sized threatened species were detected in dingo scats (because most, if not all of these are already locally extinct [11], [13], [51]), where small plains mice, fawn hopping-mice and dusky hopping-mice were the only listed threatened species detected at our study sites (Table 4).

### Plains mice

**Figure 2:**
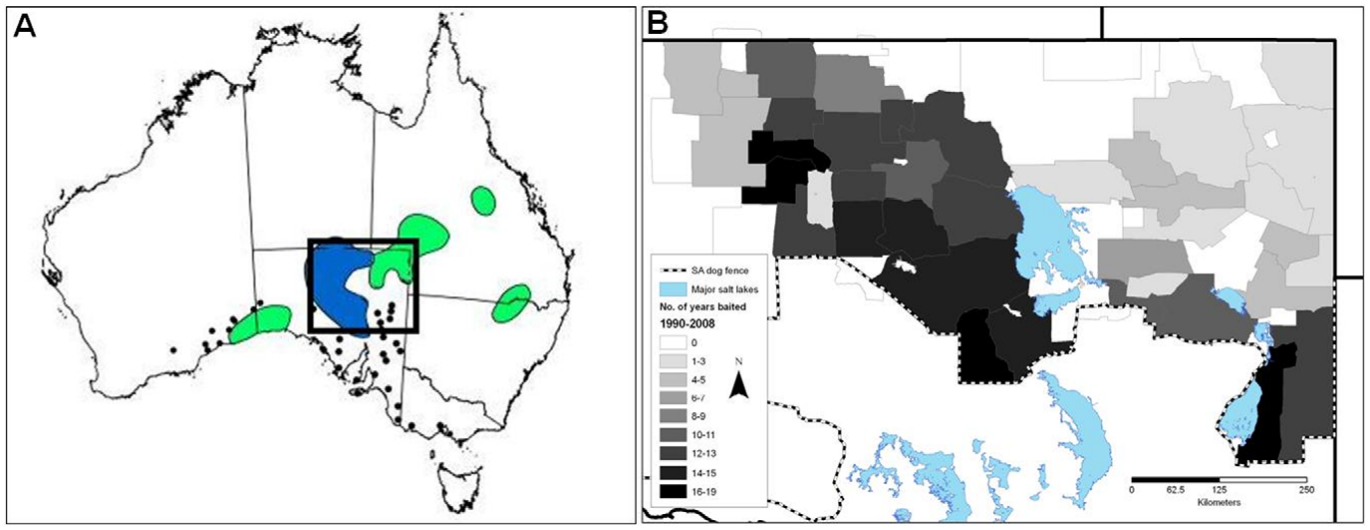
The (A) historical (pre-1980; green) and extant (post-1980; blue) distribution of plains mice *Pseudomys australis* and the location of sub-fossil bone material (black dots) (from [**54**]), and the (B) frequency and distribution of dingo control in northern South Australia 1990–2008 (from [**38**]).

Plains mice distribution has largely contracted to central South Australia (Fig. 2) where dingoes (and probably foxes as well) have been most heavily subjected to lethal control (Fig. 2; but see [38] for details), and they were thought to be locally extinct from the north-east of the state for several decades before their recent discovery in a dingo scat from Quinyambie [3]. The present distribution of plains mice also correlates positively with their preferred habitats (cracking clay soils) [54]. Thus, although several factors undoubtedly influence the persistence of plains mice, the geographic pattern of decline is consistent with predictions of dingo predation risk [7]. The correlation between dingo control effort and plains mice persistence suggests that dingoes may suppress plains mice similar to other arid zone rodents (e.g. [31], [33]), or that dingo control benefits plains mice as it does for some larger bodied species. In no way does this diminish the importance of other processes also threatening plains mice. Though it is tempting to conclude that the infrequency of this species in scats (Table 2) eliminates the possibility that dingoes may threaten them, detecting a prey species in dingo scats would not be expected if dingoes had already contributed to their local extinction. Moreover, given dingoes ability to exploit rodents, their absence in scats may simply mean that alternative species more preferred by dingoes (such as rabbits, kangaroos and other rodents) were available and preferentially eaten at the time (Table 2).

### Hopping-mice

The majority of fawn hopping-mice were detected in dingo scats from Cordillo Downs [3] where a relatively wide variety of other species were also consumed by dingoes (Table 2). The presence of an apparently diverse fauna assemblage is likely to moderate the effects of dingo predation [31], [55], [56] on hopping-mice populations there because dingoes may have greater opportunity to switch between prey as one species or another becomes unavailable. But at Quinyambie, where dingoes were heavily reliant only on rabbits (Table 2), the risk of hyperpredation [57], [58] on dusky hopping-mice by dingoes may be severe. Using both scat data (Table 2) and dingo density data [47] collected at the site, the hypothetical dingo predation rates of hopping-mice suggests that dingoes alone have the capacity to rapidly exterminate local populations of them under certain conditions [59].

To illustrate this, if one scat represents the prey eaten by an individual dingo in the previous 24 hours, then 8% occurrence represents at least 8 hopping-mice every 100 days per dingo per year, or 20 hopping-mice per dingo per year. Given there was a conservative average of 10 dingoes/25 km^2^ at this site at the time [47], 8% occurrence in scats could represent predation of 292 individual hopping-mice within the average home range of a dingo pack, or 12 hopping-mice/km^2^/year. However, this simple calculation assumes that the presence of hopping-mice remains in a dingo scat represents only one individual. That 63% of the scats containing hopping-mice showed them to be the sole mammalian prey detected implies that some dingoes may consume up to 20 hopping-mice/day to meet their daily energy demands of ∼1000 g of meat per day ([31], [60]; but see also [61]). This could potentially represent as many as 19 hopping-mice/km^2^/month. Thus, when deteriorating environmental conditions reduce the breeding success of hopping-mice (a relatively common occurrence in stochastic arid environments; [32], [34], [59]), dingoes alone have the theoretical capacity to force hopping-mice populations to extinction if they cannot sustain the loss of 19 individuals per km^2^ each month. If a population declined to 60 hopping-mice/km^2^ (or 10% of their peak densities recorded in comparable habitats; [62]), then a hopping-mice population may be threatened with extinction by dingo predation alone in just three months.

So how do some hopping-mice populations persist in the presence of high dingo densities when the potential risks are so severe? First, the predation rates calculated here assume that dingo population densities and predation rates remain constant as climatic conditions deteriorate and prey species decline, which is not likely. Behavioural observations of dingoes during the study [63] concur with others [59] that able dingoes may migrate to areas with higher prey availability during chronic food shortages, leaving remaining individuals to consume whatever they can find or catch, before finally scavenging carrion and then eating each other. Dingoes can disperse over 1300 km in four months or over 550 km from their point of origin in 31 days [64]. Emigration is likely coupled with increased home range sizes of remaining dingoes as the prey resources within the home range decline [65], both processes acting to reduce predation rates on dwindling hopping-mice populations. Such a survival strategy by dingoes may prevent both dingo and hopping-mice populations from local extinction in the short term [59]. This suggests that at least part of the reason why some hopping-mice populations survive (and sometimes thrive) in the presence of dingoes may not be because dingoes provide indirect benefits to them (as proposed in [66]), but because dingo predation pressure is alleviated during high-risk times when hopping-mice populations are low.

### General considerations

Threatened species were typically found infrequently in dingo scats from our sites, consistent with the findings of similar studies (e.g. [4]–[6]). Although it is tempting to view such results as evidence that dingoes do not present significant risks to threatened species, there are several important reasons why they should not be casually dismissed in this way. Dingoes probably select prey on the basis of the relative profitability of capturing and consuming one species over another [16], [31], [34], where ‘profitability’ is a function of several factors. Prey-based factors include their availability, body size, fitness, catchability and their behavioural response to the predator. Predator-based factors include their group size, social status, search image and hunting experience.

Prey availability is often unknown but particularly important, because predators would not be expected to eat a species that is not there, which means that the absence of a given species in predator scats may not be a useful indicator of the risk predators pose to them [7]. For example, brushtail possums *Trichosurus vulpecula* are detected rarely in modern dingo scats from central Australia where dingoes have been implicated in their local extinction [17]. Likewise, a study that released dingoes to eradicate feral goats *Capra hircus* from an offshore island understandably showed a decline in the presence of goats in dingo scats as dingoes eliminated them, where subsequent scat surveys detected no goats in scats [8]; the absence of goats in later scat surveys obviously could not be used to assume that dingoes were not a threat to goats. Threatened species such as western barred bandicoots *Perameles bougainville*, numbats *Myrmecobius fasciatus* or greater stick-nest rats *Leporillus conditor* (and many others) are not found in cat, fox or dingo scats from the arid zone either (e.g. [4], [33]–[35]), for the same reasons. These examples highlight why the absence of a particular prey species should not immediately be presumed to reflect the inability of a predator to exploit them. As illustrated above by the consideration of information additional to the prevalence of plains mice and hopping-mice in dingo scats, casually dismissing the infrequent occurrence of a species in predator scats may overlook potentially important factors limiting the recovery of some threatened species.

The availability of alternative prey may also be a particularly important consideration. Without knowledge of the alternative species available to predators it is impossible to determine their preference for one species over another. Species detected frequently in predator scats therefore represent the selection of that species from the suite of species available to them at that time and place; they do not represent the potential effects of predators on prey in a different context [8]. For example, rabbits and rodents were detected relatively infrequently in dingo scats from the north-western sites but were staple prey at the north-eastern sites (Table 2), indicating that dingoes will frequently eat these prey in some circumstances. Also, plains mice were understandably not detected in dingo scats from sites where plains mice persist, where alternative preferred prey (e.g. kangaroos and other rodents) are apparently more common (e.g. [42]) and were more frequently consumed (Table 2).

Although dingoes and the three threatened rodents detected in their scats coexisted sympatrically prior to European settlement, they did not do so in the presence of rabbits, livestock or other landscape-changing effects of pastoralism [9], [67], [68]. Though robust data on dingo densities was not collected at the time, post-European provision of virtually unlimited prey and water resources has undoubtedly increased the range and population densities of dingoes in areas outside the dog fence (e.g. [16], [33], [69]). Thus, these threatened rodents have not been exposed to such high and ubiquitous densities of dingoes until modern times. Put simply, the ecological circumstances have changed significantly since dingoes, native rodents and other now-threatened species coexisted sustainably [21]. The theoretical capacity for dingoes to locally depopulate rodent species is disconcerting given the restricted distribution of many arid-zone rodents [11], [70]. However, dingo predation of many rodents is undoubtedly sustainable during ‘boom’ times, and it is the ‘bust’ times that are of most concern [32], [59], [71]. In our efforts to assist the recovery of threatened species, we are largely unable to manipulate rainfall and vegetation growth, but we have some degree of ability to manage dingoes (and other predators) through the use of lethal and non-lethal control techniques [22], [72].

This study has shown that dingoes eat a wide variety of prey items in northern South Australia and that the remains of small and threatened species typically occurred infrequently in dingo scats. Importantly however, consideration of these results in light of additional information on dingo control effort and predation rates suggests that dingoes have the potential capacity to exterminate or suppress local populations of rodents under certain conditions, and that dingo control may benefit small mammals as it does for some larger-bodied species. Whether or not this occurs in reality likely depends on a range of complex ecological interactions specific to the site or population of interest. Thus, the data presented here cannot demonstrate that dingoes *do* present a risk to rodent populations, but rather suggests that they *could* under certain conditions. The direct effects of dingoes on small and threatened prey species therefore warrant specific investigation before dingo populations are permitted to increase in areas with species of conservation concern. Such studies may include assessment of the spatial, numerical and functional relationships between dingoes and rodents over time, inclusive of information on the prevalence of threatened species in dingo scats during periods of prey population declines. The effects of dingo control on threatened fauna also require urgent attention [7], [21]. While the limitations and uses of predator scat data have been discussed for dingoes and rodents, these principles may be widely applicable to studies of predator risks to threatened species in many other places.

## Supporting Information

Table S1 **Studies reporting the collection of dingo scats or stomach contents.**
(DOC)

Table S2 **The frequency distribution of reported sample sizes in 47 studies of dingo scats and stomachs from six climate types across Australia.**
(XLSX)
