# Supplementary material for: Assessing Predation Risk to Threatened Fauna from their Prevalence in Predator Scats: Dingoes and Rodents in Arid Australia
Source: PLoS One. 2012 May 1;7(5):e36426. doi: 10.1371/journal.pone.0036426 (PMC3341367; doi:10.1371/journal.pone.0036426)
Supplement: Table S1 — Studies reporting the collection of dingo scats or stomach contents. (DOC) [file pone.0036426.s001.doc]

**Table S1 – Studies reporting the collection of dingo scats or stomach contents**

|  | **Study** | **Sample size** | **Period** | **Major sites** | **Location** | **Climate** |
| --- | --- | --- | --- | --- | --- | --- |
| 1 | Burnett 1995 | 282 | 4 yrs | 1 | Wet tropics, NE QLD | Wet tropical |
| 2 | Vernes 2000 | 51 | 2 yrs | 1 | Wet tropics, NE QLD | Wet tropical |
| 3 | Vernes et al. 2001 | 383 | 8 yrs | 1 | Wet tropics, NE QLD | Wet tropical |
| 4 | Brook and Kutt 2011 | 178 | 6 yrs | Various | NE QLD | Tropical |
| 5 | Byrne 2009 | 133 | 1 yr | 1 | Kimberley, WA | Tropical |
| 6 | Corbett 1995^ | 6,722 | 7 yrs | 1 | Kapalga, NT | Tropical |
| 7 | Augusteyn 2010 | 179 | 2 yrs | 1 | Taunton NP, central QLD | Sub-tropical |
| 8 | Pavlov and Heise 1998 | 332 | 2 yrs | 1 | Shoalwater Bay, central QLD | Sub-tropical |
| 9 | Allen and Gonzalez 2000 | 147 | 1 yr | 1 | Shoalwater Bay, central QLD | Sub-tropical |
| 10 | Allen et al. 1998 | 192 | 3 yrs | 1 | Townshend Island, off central QLD | Sub-tropical |
| 11 | Twyford 1995 | 1,356 | 2 yrs | 2 | Fraser Island, Cooloola NP | Sub-tropical |
| 12 | Baker In prep | 163 | 2 yrs | 1 | Fraser Island | Sub-tropical |
| 13 | Banks et al. 2003 | 4 | Once-off | 1 | Central QLD | Semi-arid |
| 14 | Allen 2005 | 1,040 | 5 yrs | 2 | SW QLD, Gulf of Carpentaria | Semi arid, Tropical |
| 15 | Robertshaw and Harden 1985^ | 1,993 | 5 yrs | 1 | NE NSW | Temperate |
| 16 | Fleming et al. In prep | 172 | 2 yrs | 1 | NE NSW | Temperate |
| 17 | Lunney et al. 1996 | 164 | 0.5 yrs | 1 | NE NSW | Temperate |
| 18 | Glen and Dickman 2008 | 73 | 2 yrs | 1 | NE NSW | Temperate |
| 19 | Glen et al. 2011 | 68 | 2 yrs | 1 | NE NSW | Temperate |
| 20 | G. Ballard, unpublished data | >2,000 | 5 yrs | 1 | NE NSW | Temperate |
| 21 | Mitchell and Banks 2005 | 264 | 0.5 yrs | 1 | Blue Mountains | Temperate |
| 22 | Purcell 2009 | 1,489 | 2 yrs | 1 | Blue Mountains | Temperate |
| 23 | Pascoe 2011 | 451 | 2 yrs | 1 | Blue Mountains | Temperate |
| 24 | Lunney et al. 1990^ | 1,085 | 1 yr | 1 | SE NSW | Temperate |
| 25 | Newsome et al. 1983a^ | 1,416 | 9 yrs | 2 | SE NSW | Temperate |
| 26 | Meek and Triggs 1998 | 29 | 5 yrs | 1 | SE NSW | Temperate |
| 27 | Claridge et al. 2010 | 129 | 5 yrs | 1 | SE NSW | Temperate |
| 28 | Newsome et al. 1983b^ | 530 | 6 yrs | 1 | SE NSW/NE VIC | Temperate |
| 29 | Brown and Triggs 1990 | 828 | 4 | 1 | NE VIC | Temperate |
| 30 | Coman 1972^ | 166 | 2 yrs | 1 | VIC | Temperate |
| 31 | Triggs et al. 1984^ | 412 | 2 yrs | 1 | SE VIC | Temperate |
| 32 | Wallach and O'Neill 2008 | 20 | Once-off | 1 | Sthn SA | Temperate |
| 33 | Wallach et al. 2009 | 890 | 3 yrs | 5 | Nthn SA | Arid |
| 34 | Moseby et al. 1998 | 26 | 1 yr | 1 | Nthn SA | Arid |
| 35 | Allen and Leung 2012 | 4,087 | 4 yrs | 3 | Strzelecki, Sturt & Pedirka Deserts | Arid |
| 36 | Cupples et al. 2011* | 232 | Once-off | 3 | Nullarbor Plain, Simpson & Strzelecki Deserts | Arid |
| 37 | Marsack and Campbell 1990^ | 131 | 4 yrs | 1 | Nullarbor Plain | Arid |
| 38 | Letnic et al. 2009* | 451 | 3 yrs | Various | Along the dingo barrier fence | Arid |
| 39 | Pavey et al. 2008 | 316 | 2 yrs | 1 | Simpson Desert | Arid |
| 40 | Foulkes 2001 | 310 | 3 yrs | 1 | Alice Springs region | Arid |
| 41 | Corbett and Newsome 1987^ | 285 | 6 yrs | 1 | Sth of Alice Springs | Arid |
| 42 | Eldridge et al. 2002 | 763 | 3 yrs | 1 | Sth of Alice Springs | Arid |
| 43 | Lundie-Jenkins et al. 1993 | 58 | 1.5 yrs | 1 | Tanami Desert | Arid |
| 44 | Paltridge 2002 | 75 | 2 yrs | 1 | Tanami Desert | Arid |
| 45 | Newsome 2011 | 1,907 | 2 yrs | 1 | Tanami Desert | Arid |
| 46 | Thomson 1992^ | 471 | 7 yrs | 1 | Pilbara, WA | Semi-arid |
| 47 | Whitehouse 1977^ | 160 | 5 yrs | Various | Rangelands, WA | Semi-arid |
|  | ***TOTAL****†* | **19,242** |  |  |  |  |

**Records from these studies may overlap. ^Results of these studies are summarised in Chapter 7 of Corbett (2001). †Excluding the 12,802 records from studies summarised in Corbett (2001).*

**References**

Allen B. L., Leung L. K.-P. (2012) Assessing predation risk to threatened fauna from their prevalence in predator scats: dingoes and rodents in arid Australia. *PLoS ONE* **xx**, xx, xx-xx.

Allen L., Lee J., Gonzalez A. (1998) 'The management and eradication of feral goats from Townshend Island, Final report to Department of Defence.' (Department of Natural Resources: Toowoomba)

Allen L. R. (2005) The impact of wild dog predation and wild dog control on beef cattle production, PhD Thesis. Department of Zoology, The University of Queensland.

Allen L. R., Gonzalez A. (2000) 'Movement of dingoes from Shoalwater Bay training area. Final report to Department of Defence.' (Department of Natural Resources: Toowoomba)

Augusteyn J. (2010) Determining the effectiveness of canine control at Taunton National park (Scientific) and its impact on the population of bridled nailtail wallabies In 'Proceedings of the Queensland Pest Animal Symposium'. Gladstone, Queensland.

Baker N. (In prep) 'The ecology of the dingo on Fraser Island: understanding complex interactions using non-invasive techniques.' (School of Animal Studies, The University of Queensland: Gatton)

Banks S. C., Horsup A., Wilton A. N., Taylor A. C. (2003) Genetic marker investigation of the source and impact of predation on a highly endangered species. *Molecular Ecology* **12**, 6, 1663-1667.

Brook L. A., Kutt A. S. (2011) The diet of the dingo (*Canis lupus dingo*) in north-eastern Australia with comments on its conservation implications. *The Rangeland Journal* **33**, 79–85.

Brown G. W., Triggs B. E. (1990) Diets of wild canids and foxes in East Gippsland 1983-1987, using predator scat analysis. *Australian Mammalogy* **13**, 209-213.

Burnett S. (1995) 'Project Gondwana: The distribution and conservation significance of dingoes, foxes, and feral cats in the uplands of the Wet Tropics World Heritage Area.' (Department of Tropical Environment Studies and Geography, and Zoology Department, James Cook University: Townsville)

Byrne S. (2009) 'Relationships between dingoes and their prey in northern Australia. Industrial placement report ENVM 3521.' (The University of Queensland and the Australian Wildlife Conservancy: Brisbane)

Claridge A. W., Mills D. J., Barry S. C. (2010) Prevalence of threatened native species in canid scats from coastal and near-coastal landscapes in south-eastern Australia. *Australian Mammalogy* **32**, 2, 117-126.

Coman B. J. (1972) Helminth parasites of the dingo and feral dog in Victoria with some notes on the diet of the host. *Australian Veterinary Journal* **48**, 8, 456-461.

Corbett L. (1995) Does dingo predation or buffalo competition regulate feral pig populations in the Australian wet-dry tropics? An experimental study. *Wildlife Research* **22**, 65-74.

Corbett L., Newsome A. E. (1987) The feeding ecology of the dingo. III. Dietary relationships with widely fluctuating prey populations in arid Australia: an hypothesis of alternation of predation. *Oecologia* **74**, 215-227.

Corbett L. K. (2001) 'The dingo in Australia and Asia (Second edn).' (J.B. Books, South Australia: Marleston)

Cupples J. B., Crowther M. S., Story G., Letnic M. (2011) Dietary overlap and prey selectivity among sympatric carnivores: could dingoes suppress foxes through competition for prey? *Journal of Mammalogy* **92**, 3, 590-600.

Eldridge S. R., Shakeshaft B. J., Nano T. J. (2002) 'The impact of wild dog control on cattle, native and introduced herbivores and introduced predators in central Australia, Final report to the Bureau of Rural Sciences.' Parks and Wildlife Commission of the Northern Territory, Alice Springs.

Fleming P. J. S., Thompson J. A., Kamphorst P., Jarman P. J. (In prep) Does control of wild canids effect the abundance of their prey? *Unpublished manuscript* **xx**, x, xx-xx.

Foulkes J. N. (2001) The ecology and management of the common brushtail possum *Trichosaurus vulpecula* in central Australia. *PhD thesis*, The University of Canberra.

Glen A. S., Dickman C. R. (2008) Niche overlap between marsupial and eutherian carnivores: Does competition threaten the endangered spotted-tailed quoll? *Journal of Applied Ecology* **45**, 2, 700-707.

Glen A. S., Pennay M., Dickman C. R., Wintle B. A., Firestone K. B. (2011) Diets of sympatric native and introduced carnivores in the Barrington Tops, eastern Australia. *Austral Ecology* **36**, 3, 290-296.

Letnic M., Koch F., Gordon C., Crowther M., Dickman C. (2009) Keystone effects of an alien top-predator stem extinctions of native mammals. *Proceedings of the Royal Society of London B* **276**, 3249-3256.

Lundie-Jenkins G., Corbett L. K., Phillips C. M. (1993) Ecology of the rufous hare-wallaby, *Lagorchestes hirsutus* Gould (Marsupialia: Macropodidae), in the Tanami Desert, Northern Territory. III. Interactions with introduced mammal species. *Wildlife Research* **20**, 495-511.

Lunney D., Law B., Rummery C. (1996) Contrast between visible abundance of the brush-tailed rock wallaby, *Petrogale penicillata*, and its rarity in fox and dog scats in the gorges east of Armidale, New South Wales. *Wildlife Research* **23**, 3, 373-380.

Lunney D., Triggs B., Eby P., Ashby B. (1990) Analysis of scats of dogs Canis familiaris and foxes Vulpes vulpes (Canidae: Carnivora) in coastal forests near Bega, New South Wales. *Australian Wildlife Research* **17**, 61-68.

Marsack P., Campbell G. (1990) Feeding behaviour and diet of dingoes in the Nullarbor region, Western Australia. *Australian Wildlife Research* **17**, 349-357.

Meek P. D., Triggs B. (1998) The food of foxes, dogs and cats on two peninsulas in Jervis Bay, New South Wales. *Proceedings of the Linnean Society of New South Wales* **120**, 117-127.

Mitchell B. D., Banks P. B. (2005) Do wild dogs exclude foxes? Evidence for competition from dietary and spatial overlaps. *Austral Ecology* **30**, 5, 581-591.

Moseby K., Read J., Gee P., Gee I. (1998) A study of the Davenport Range black-footed rock wallaby colony and possible threatening processes. *Final report to Wildlife Conservation Fund, Department for Environment and Heritage*, Adelaide.

Newsome A. E., Catling P. C., Corbett L. K. (1983a) The feeding ecology of the dingo. II. Dietary and numerical relationships with fluctuating prey populations in south-eastern Australia. *Australian Journal of Ecology* **8**, 345-366.

Newsome A. E., Corbett L. K., Catling P. C., Burt R. J. (1983b) The feeding ecology of the dingo. I. Stomach contents from trapping in south-eastern Australia, and the non-target wildlife also caught in traps. *Australian Wildlife Research* **10**, 3, 477-486.

Newsome T. M. (2011) Ecology of the dingo (*Canis lupus dingo*) in the Tanami Desert in relation to human-resource subsidies. *PhD thesis*, The University of Sydney.

Paltridge R. (2002) The diets of cats, foxes and dingoes in relation to prey availability in the Tanami Desert, Northern Territory. *Wildlife Research* **29**, 389-403.

Pascoe J. H. (2011) Apex predators in the Greater Blue Mountains World Heritage Area. *PhD thesis*, The University of Western Sydney.

Pavey C. R., Eldridge S. R., Heywood M. (2008) Population dynamics and prey selection of native and introduced predators during a rodent outbreak in arid Australia. *Journal of Mammalogy* **89**, 3, 674-683.

Pavlov P. M., Heise S. R. (1998) 'Dingo/feral animal management – Shoalwater Bay Training Area. Report to Department of Defence.' (Department of Natural Resources: Toowoomba)

Purcell B. V. (2009) Order in the pack: Ecology of *Canis lupus dingo* in the southern Greater Blue Mountains World Heritage Area, PhD Thesis. University of Western Sydney, School of Natural Sciences.

Robertshaw J. D., Harden R. H. (1985) The ecology of the dingo in north-eastern New South Wales, II. Diet. *Australian Wildlife Research* **12**, 1, 39-50.

Thomson P. C. (1992) The behavioural ecology of dingoes in north-western Australia: III. Hunting and feeding behaviour, and diet. *Wildlife Research* **19**, 5, 531-541.

Triggs B., Brunner H., Cullen J. M. (1984) The food of fox, dog and cat in Croajingalong National Park, south-eastern Victoria. *Australian Wildlife Research* **11**, 491-499.

Twyford K. (1995) 'Investigations into the dietary ecology of dingoes on Fraser Island. Third Interim Report.' (Queensland Parks and Wildlife Service: Fraser Island)

Vernes K. (2000) Immediate effects of fire on survivorship of the northern bettong (*Bettongia tropica*): an endangered Australian marsupial. *Biological Conservation* **96**, 3, 305-309.

Vernes K., Dennis A., Winter J. (2001) Mammalian diet and broad hunting strategy of the dingo (*Canis familiaris dingo*) in the wet tropical rain forests of northeastern Australia. *Biotropica* **33**, 2, 339-345.

Wallach A. D., O'Neill A. J. (2008) 'Persistence of endangered species: Is the dingo the key?' (Final Report to Department of Environment and Heritage Wildlife Conservation Fund (National Parks and Wildlife SA)

Wallach A. D., Ritchie E. G., Read J., O'Neill A. J. (2009) More than mere numbers: The impact of lethal control on the stability of a top-order predator. *PLoS ONE* **4**, 9, e6861.

Whitehouse S. J. O. (1977) The diet of the dingo in Western Australia. *Australian Wildlife Research* **4**, 145-150.
